# The impact of Bruton’s tyrosine kinase inhibitor treatment on COVID-19 outcomes in Chinese patients with chronic lymphocytic leukemia

**DOI:** 10.3389/fonc.2024.1396913

**Published:** 2024-05-21

**Authors:** Shenmiao Yang, Rong Wei, Hongxia Shi, Yazhe Wang, Yueyun Lai, Xiaosu Zhao, Jin Lu, Norbert Schmitz

**Affiliations:** ^1^ Peking University Institute of Hematology, Peking University Peoples’ Hospital, Beijing, China; ^2^ Department of Medicine A, University Hospital Muenster, Muenster, Germany

**Keywords:** BTK inhibitors, COVID-19, chronic lymphocytic leukemia, SARS-CoV-2, anti CLL treatment

## Abstract

**Background:**

Impact of B-cell depletion following treatment with Bruton tyrosine kinase-inhibitors (BTKi) on the outcome of SARS-CoV-2 infection in chronic lymphocytic leukemia (CLL) patients remain controversial. We investigated the impact of BTKi on susceptibility and the severity of COVID-19 in Chinese patients with CLL during the first wave of COVID-19 (Omicron variant).

**Methods:**

CLL patients (n=171) visiting the Institute of Hematology, Peoples’ Hospital, China (November 15, 2022- January 20, 2023) were included in the study. Seventeen patients receiving BTKi and venetoclax with or without obinutuzumab were excluded. Data from 117 patients receiving treatment with BTKi were collected using a standardized questionnaire through telephone interviews. Thirty-four patients without CLL-specific treatment served as controls. The data was analysed using IBM SPSS Software version 21 and a P value of <0.05 was considered statistically significant.

**Results:**

The median age of patients was 67 years and majority were males (n=100). Treatment with BTKi was not associated with higher incidence of COVID-19 (74% [95% Confidence Interval (CI) 60%, 92%]) versus 74% (CI 48%, 100%) without any treatment (*P*=0.92). Hypoxemia was reported by 45% (32%, 61%) and 16% (4%, 41%) (*P*=0.01). BTKi was the only independent risk factor of hypoxemia (Hazard Ratio [HR], 4.22 [1.32, 13.50]; *P* = 0.02). Five (5.7%) patients with COVID-19 under BTKi required ICU admission; 4 of them died. No ICU admissions/deaths were observed in the control group.

**Conclusion:**

In Chinese patients with CLL and treated with BTKi experienced more severe lung disease and ICU admissions due to COVID-19 than patients without CLL therapy. Frequency of infections with SARS-CoV-2, however, was not different in patients with or without BTKi treatment.

## 1 Introduction

In May 2023, the World Health Organization (WHO) declared Coronavirus disease 2019 (COVID-19) caused by severe acute respiratory syndrome coronavirus 2 (SARS-CoV-2) was no longer a “global health emergency.” (1, 2) Unlike other variants of SARS-CoV-2 that caused substantial morbidity and mortality, Omicron variant was more transmissible, mostly causing less severe clinical symptoms. Importantly, Omicron fails to elicit neutralizing antibody responses generated by vaccination or previous SARS-CoV-2 infections (3, 4). The “zero COVID policy” introduced in China shortly after the first cases of COVID-19 occurred refers to strict massive community-based screening, contact tracing, quarantine and isolation of SARS-CoV-2-positive patients as well as frequent local lockdowns to quickly break the chain of transmission and contain the spread of outbreaks (5). The Zero-COVID policy which resulted in Chinese people largely escaping from contacts with early viral variants Alpha, Beta, Gamma, and Delta and subsequent clinical symptoms was abandoned on December 7, 2022. As a consequence, it was estimated that in major cities like Beijing 60-80% of the population were infected with Omicron within the next 1.5 months (6). The Omicron variants BF.7, BA.5.1, and BA.5.2 became predominant with a significant resurgence of the BF.7 variant since late September 2022 (7, 8).

Realizing the specific epidemiological background we wanted to investigate which consequences the sudden exposure to SARS-CoV-2 might have in patients with chronic lymphocytic leukemia (CLL) under treatment with BTKi and compare our results with those from Western countries reporting on the role of BTKi on CLL patients with COVID-19 (9–11). BTKis are recommended as frontline therapy for CLL due to their superior progression-free survival (PFS) outcomes compared to the conventional chemotherapy (12–17). Anti-inflammatory activity of BTKi was reported to protect patients from the cytokine release syndrome observed in patients with SARS-CoV-2 infection (18). Also, CLL patients with COVID-19 under BTKis appear to have minimal oxygen requirements, mechanical ventilation and shorter hospital stays (9, 10). On the other hand, BTKi-related immunosuppression seemed to cause a high risk of infection and delayed virus clearance (19, 20). Whether continuation of BTKi treatment represents a risk factor for CLL patients to experience COVID-19, remains an open question particularly in patients not previously exposed to the virus. In the most recent consensus, the European Society for Medical Oncology (ESMO) and the European Hematology Association (EHA) recommended deferring the commencement of treatment with BTKi until nasopharyngeal swab negativity and resolution of clinical symptoms. Targeted therapies should be put on hold until these goals were achieved (21). Following these recommendations, however, may influence the course of disease as the interruption of BTKi therapy has been associated with inferior progression-free survival and a higher risk of Richter’s transformation (22).

## 2 Methods

### 2.1 Study setting and participants

This single-center, retrospective study was conducted in CLL patients who were diagnosed and regularly followed at peoples’ hospital, Peking University, Beijing, China. The institute of Hematology is a tertiary referral center serving the Beijing area with 21 million inhabitants but additionally takes care of frequent referrals from all over China. All patient data collected for this analysis were obtained through telephone interviews thereby adhering to strict local safety measures in place between March 15, to April 1, 2023, in order to minimize the risk of exposure to SARS-CoV-2 for both patients and healthcare providers.

The study was reviewed and approved by the Ethics Committee of Peking University Peoples’ hospital (No.2023PHB015-001), and was conducted in accordance with the Declaration of Helsinki. Informed consent was obtained from all patients before inclusion in the study. Patient confidentiality was maintained throughout the research process, all data were anonymized and securely stored.

### 2.2 Data collection and management

Patient data were collected through structured telephone interviews conducted by trained healthcare professionals. A standardized questionnaire was used to obtain information regarding demographics, COVID-19 occurrence and outcome, vaccination and CLL status, comorbidities, treatment history, BTKi usage, and antiviral therapy (see **Supplementary 1**). All data obtained during the interviews were recorded and stored using a secure electronic database. Patient confidentiality and data protection were ensured throughout the study.

COVID-19 was diagnosed in patients with any symptom of respiratory infection and laboratory evidence of SARS-CoV-2, including either a positive nucleic acid PCR or a positive antigen detection. Severe COVID-19 was defined as the occurrence of any of the following: dyspnea, a respiratory rate of ≥30/min, blood oxygen saturation of ≤93%, a ratio of partial pressure of arterial oxygen to the fraction of inspired oxygen (PaO2:FIO2) <300 mm Hg, or pulmonary infiltrates covering >50% of the lungs (23). All patients or their caregivers reported the oxygen saturation with home oxygen monitors.

### 2.3 Statistics

Continuous variables are presented as median, interquartile range [IQR], or range, whereas categorical variables are presented as frequencies and percentages. Chi-square test was used to determine the association between BTKi treatment and the occurrence of COVID-19 as well as severe manifestations such as hypoxemia, ICU admission, and mortality. Kaplan-Meier survival curves were used to depict the impact of BTKi usage on patient survival. Univariate and multivariate analyses were performed using logistic regression to identify independent risk factors for severe COVID-19 infections. Hazard ratios (HR) with 95% confidence intervals (CI) and P- values for the effect of each variable on COVID-19 outcomes were estimated. Mann-Whitney U test was used to compare durations of BTKi interruption and previous BTKi exposure between the patients with and without progression after cessation of treatment with BTKi. A two‐sided P value of <0.05 was considered statistically significant. All statistical analyses were performed using IBM SPSS Software version 21.

## 3 Results

### 3.1 Patient characteristics

Overall, 171 CLL patients visited the outpatient department of peoples` hospital from November 15, 2022, to January 20, 2023, and were included in the study. These patients were interviewed from March 15, 2023, to April 1, 2023. 168 patients and their caregivers coming from 17 provinces all over China responded to the interview by completing the questionnaires between March 15 and April 1, 2023. Ninety-six patients stayed in Beijing during the pandemic, the other patients travelled back to their hometowns after visiting the outpatient department. Seventeen patients receiving BTKi and venetoclax with or without obinutuzumab were excluded from this analysis; 151 patients remained on study. The majority of patients were male (n=100; 66%), their median age was 67 years (range 36-87 years) (**Table 1**) A total of 117 patients (78%) were treated with BTKi. Ibrutinib was received by 68 patients (58%) followed by zanubrutinib (n=30; 26%), acalabrutinib (n=11; 9%) and orelabrutinib (n=8; 7%). The median duration of exposure to BTKi varied across the different treatments. The longest duration of exposure was observed for orelabrutinib (46 months, IQR: 30-48) followed by ibrutinib (39 months, IQR: 29-53), zanubrutinib (25 months, IQR:18-61) and acalabrutinib (20 months, IQR: 15-23). 34 patients remained off therapy during the time this study was done. 67% (67 of 117) of the patients under BTKi treatment and 65% (22 of 34) among the patients without treatment were male. Median age was 67 years (range: 36, 87 years) and 66 years (range, 40, 81) in the BTKi group and the no treatment group, respectively. 67% and 62% of patients were older than 65 years, and 15% of patients were older than 75 years in both groups. Comorbidities were comparable. The median CIRS score was 2 (range: BTKi group 0, 5; no treatment group 0, 10). Three of 117 patients experienced progressive disease while on BTKi but had not changed treatment while on study. One of 34 patients had progressive disease but without symptoms and did not meet the criteria of active CLL according to the iwCLL (24). Of 34 patients who were untreated, 17 patients (50%) were vaccinated against COVID-19, whereas in the BTKi treatment group only 21 of 117 (18%) were vaccinated (P<0.01). Chinese COVID-19 vaccines (Vero cell) (Sinopharm, Beijing Institute of Biological Products Co., Ltd and Sinovac, Sinovac Life Sciences Co., Ltd) were used in all vaccinated patients. Importantly, no patient reported any known exposure to SARS-Cov-2 or symptoms of COVID-19.

**Table 1:** Demographics and baseline clinical characteristics of the study patients.

Characteristics	Total
	N=151
Male, n (%)	100 (66)
Age, median (range), years	67 (36-87)
Age <65 years, n (%)	52 (34)
Age 65-75 years, n (%)	77 (51)
Age ≥75 years, n (%)	22 (15)
CIRS ≥7, n (%)	6 (4)
Progressive disease at COVID-19, n (%)	4 (3)
Vaccinated, n (%)	38 (25)
Median Dose of vaccines (range)	2 (1-3)
Median time between the lase dose and COVID-19, range (months)	6 (0-20)
Patients on BTKi, n (%)	117 (78)
Ibrutinib, n (%)	68 (58)
Zanubrutinib, n (%)	30 (26)
Orelabrutinib, n (%)	8 (7)
Acalabrutinib, n (%)	11 (9)
COVID-19 disease, n (%)	112 (74)
Antivirus treatment, n (%)	25 (17)

BTKi, Bruton tyrosine kinase inhibitors; CIRS, Cumulative Illness Rating Scale; COVID-19, coronavirus disease 2019; N, total number; n, number in particular category.

### 3.2 BTKi and COVID-19

A total of 112 (74.2%) patients experienced symptoms of COVID-19. Patients receiving BTKi treatment and patients without treatment exhibited similar rates of COVID-19 (BTKi group: 87 patients (74%, 95%CI [60, 92]); no treatment group: 25 patients (74%, 95% CI [48,109] [n=25], P=0.92) (**Table 2**).

**Table 2:** Comparison of risk factors between BTKi and no treatment groups.

Risk factors	BTKi N=117	No treatment N=34	P value
Male, n (%)	78 (67)	22 (65)	0.83
Age <65 years, n (%)	39 (33)	13 (38)	0.85
Age 65-75 years, n (%)	61 (52)	16 (47)	
Age ≥75 years, n (%)	17 (15)	5 (15)	
CIRS ≥7, n (%)	6 (5)	0 (0)	0.34
Progressive disease at COVID-19, n (%)	3 (3)	1 (3)	1
Doses of vaccine			
0, n (%)	96 (82)	17 (50)	<0.001
1, n (%)	6 (5)	0 (0)	
2, n (%)	5 (4)	9 (27)	
3, n (%)	10 (9)	8 (23)	
Time from the last dose of vaccine to COVID-19, median (range), months	1 (0-19)	14 (0-20)	<0.001
COVID-19 disease, n (%)	87 (74)	25 (74)	0.92
Antivirus treatment, n (%)	22/87 (25)	3/25 (12)	0.16

BTKi, Bruton tyrosine kinase inhibitors; CIRS, Cumulative Illness Rating Scale; COVID-19, coronavirus disease 2019; N, total number; n, number in particular category.

Antiviral treatment with a combination of nirmatrelvir-ritonavir (NMVr) was received by 25 of 112 (22%) COVID-19 patients, of whom only 3 patients started treatment within five days after developing first symptoms. All patients with hypoxemia started antiviral treatment only after COVID-19 was diagnosed and hypoxemia had developed.

**Figure 1:**
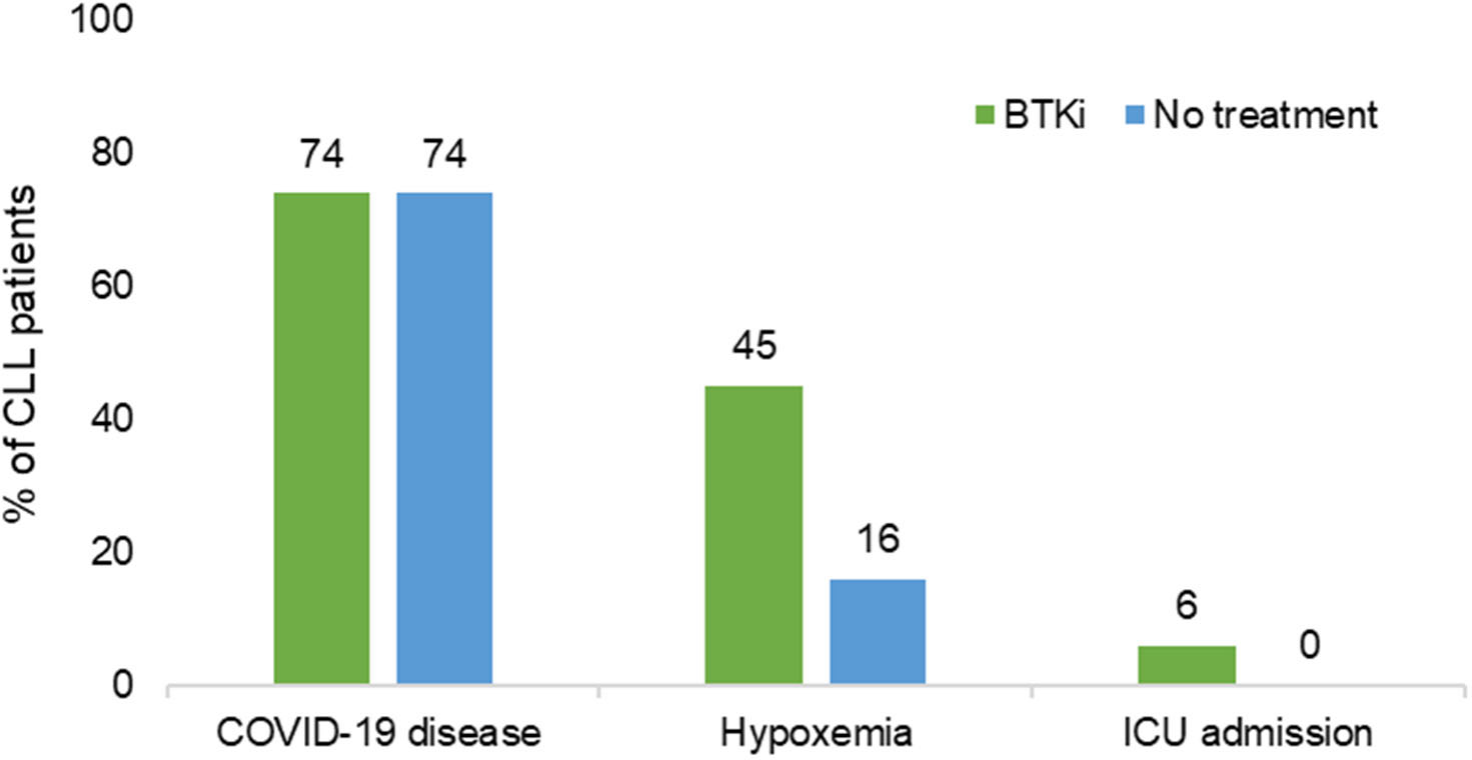
Association of BTKi with severe COVID-19. BTKi, Bruton tyrosine kinase inhibitors; ICU, intensive care unit; N, total number.

**Figure 2:**
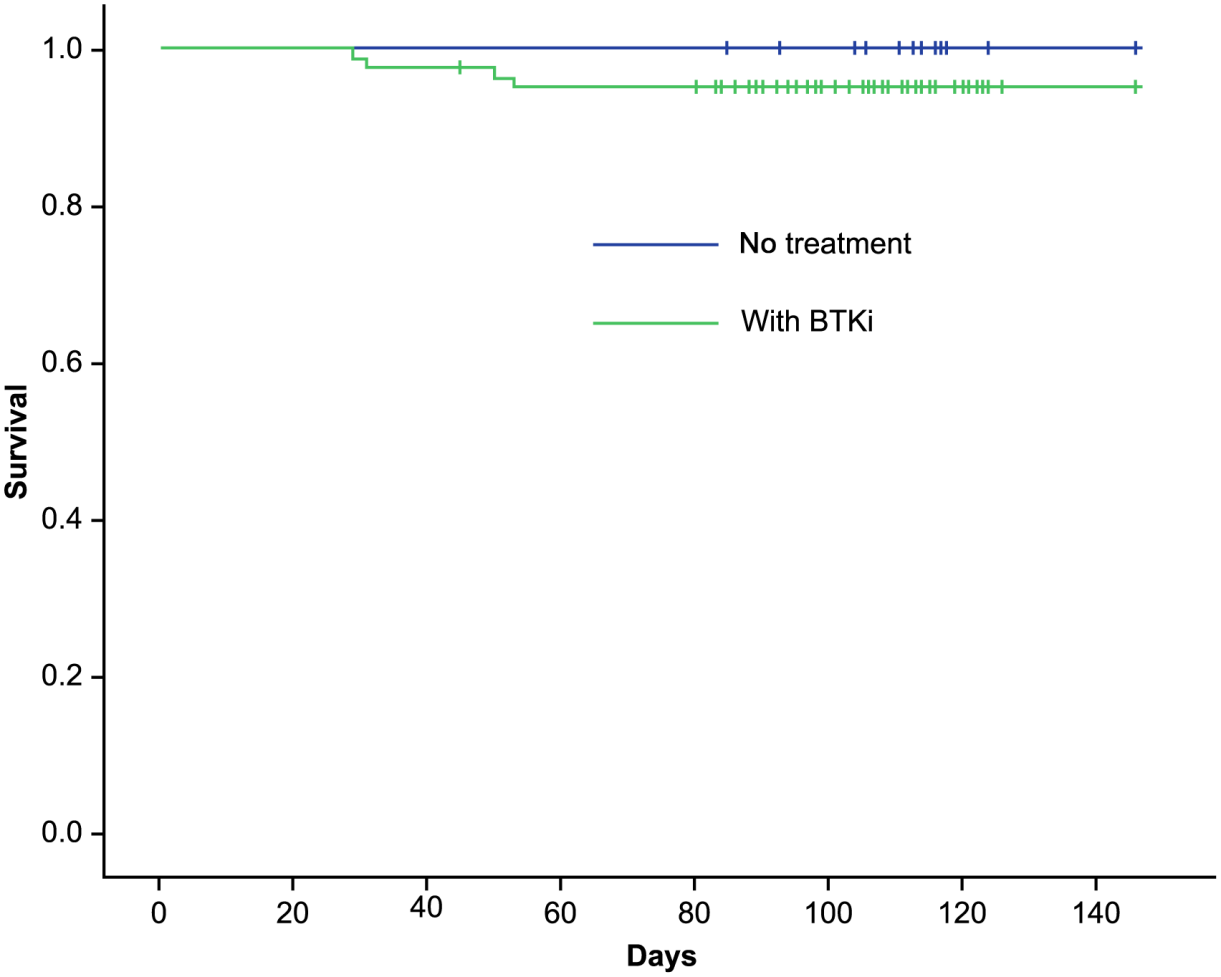
Kaplan-Meier survival curve of CLL patients with COVID-19.

A significantly higher rate of hypoxemia was observed in the BTKi group (n=39; 45%, 95%CI [32, 61]) compared to the no treatment group (n=4; 16%, 95% CI [4,41]) (P=0.01). Five (5.7%) patients with COVID-19 under BTKi needed admission to ICU; 4 of them died. Details of 4 patients who did not survive is provided in **Supplementary Table 1**. No ICU admissions and no death were seen in the “no CLL treatment” group (**Figure 1**). Overall survival of patients experiencing COVID-19 while treated or not treated with BTKi is shown in **Figure 2**.

Univariate analysis revealed that age ≥65 years (HR: 2.39; 95% CI [1.02, 5.62]; P=0.04), no vaccination (HR: 3.80; 95% CI [1.32, 10.99]; P=0.01), and treatment with BTKi (HR: 4.27; 95% CI [1.35, 13.47]; P=0.01) were significant risk factors for hypoxemia in patients with COVID-19, no significant differences were observed for sex, CIRS ≥7, and progressive versus stable disease. All factors with a P value <0.2 were included in the multivariate analysis. Continuous treatment with BTKi remained the only independent risk factor for hypoxemia in CLL patients with COVID-19 (HR: 4.22, 95% CI [1.32, 13.50]; P=0.02) (**Table 3**).

**Table 3:** Correlation analysis for risk factors associated with hypoxemia.

	Uni-variate analysis		Multi-variate analysis	
	HR [95% CI]	P value	HR [95% CI]	P value
Male	1.29 [0.56, 2.97]	0.55		
Age≥65 years	2.39 [1.02, 5.62]	0.04	2.36 [0.98, 5.58]	0.06
CIRS≥7	0 [0, -]	0.99		
Progressive disease at COVID-19	1.63 [0.22,13.47]	0.63		
Doses of vaccine	0.60 [0.39, 0.93]	0.02	0.73 [0.46, 1.17]	0.19
Time from the last dose of vaccine to COVID-19	0.92 [0.85-1.00]	0.06	1.05 [0.91-1.21]	0.53
BTKi treatment	4.27 [1.35, 13.47]	0.01	4.22 [1.32,13.50]	0.02

BTKi, Bruton tyrosine kinase inhibitors; CIRS, Cumulative Illness Rating Scale; COVID-19, coronavirus disease 2019.

### 3.3 BTKi interruptions in CLL with COVID-19

Fifty-eight of 87 patients diagnosed with COVID-19 stopped BTKi because of patients’ decisions or contraindications of ritonavir. The median duration of interruption was 13 (range: 1 to 60, IQR [6, 24]) days. Thirty of 58 patients reported no change in lymphocyte counts, 8 patients reported decreased lymphocyte counts, and 4 patients reported increased lymphocyte counts during the drug interruption. Sixteen patients were unable to report CBC counts. Eleven patients reported subjective enlargement of spleen or lymph nodes after stopping BTKi treatment. These patients reported a median interruption of 22 (range 8 to 60, IQR [16,35]) days which was significantly longer than the 9 days (1 to 50, IQR [5,21]) of interruption reported by patients without enlargement of organs (P=0.01). Previous BTKi exposure was not different between the patients with and without progression of lymphadenopathy or splenomegaly (median 32, range 10 to 63, IQR [14, 57] months vs. 33, range 4 to 104, IQR [20, 53] months, P=0.58). All patients reported increasing lymphocyte counts and shrinking lymph nodes and spleen after the BTKi was started again. No patient experienced Richter’s transformation.

## 4 Discussion

BTKi are frequently used to treat patients with CLL. Our results obtained in an environment shortly after the zero COVID policy had ended demonstrate that the incidence of COVID-19 in patients treated or not treated with BTKi was very similar; however, patients treated with BTKi had a 4-fold higher rate of severe COVID-19 compared to patients without BTKi.

Results of other studies from Western countries investigating the role of BTKi in COVID-19 patients with lymphoma have been inconsistent. The first report published in May 2020 showed that patients with Waldenström’s macroglobulinemia experienced less severe COVID-19 and less episodes of hospitalization when treated with BTKi (25). A protective role of BTKi also in CLL patients with COVID-19 was reported soon after (10). The European Research Initiative on CLL (ERIC) found that patients with severe COVID-19 under BTKi had a lower rate of hospitalization than patients treated with other drugs (26). The benefit of BTKi was considered to be attributable to the anti-inflammatory effect of the drug. A prospective phase II trial comparing Ibrutinib with placebo in hospitalized patients with a severe COVID-19 Infections did not find significant differences in percentage of patients alive and without respiratory failure through day 28 (27). Another international study involving CLL patients with COVID-19 infection showed similar mortality rates in patients with or without BTKi treatment (9). In contrast to the previous publication, ERIC recently reported that patients with COVID-19 and CLL/small lymphocytic lymphoma (SLL) and monoclonal B lymphocytosis (MBL) carry a higher risk of hospitalization as well as a higher risk of dying from the infection (28). The controversial results of both ERIC studies were explained by confounding factors such as differing transmissibility and virulence of prevalent virus variants, differences in the history of exposure to SARS-CoV-2 and differences in social support of patients during the long period of time they needed assistance.

Our results – similar frequencies but more severe COVID-19 in CLL patients on BTKi - must be seen against an epidemiologic background largely different from the situation in the Western countries: the zero COVID policy was followed by a steep wave of COVID-19 with the Omicron variant in heavily populated areas like Beijing after zero COVID was waived.

Also in Chinese patients the Omicron variant caused milder COVID-19 in healthy individuals and CLL patients with a 30-day fatality rate of <2% (29). In line with these findings, we report a very low mortality rate in CLL patients with COVID-19 although the rate of hypoxemia was as high as 45% in our cohort. This is higher than the 13% of patients with various hematologic malignancies infected with the Omicron variant who required oxygen support reported in a prospective multicenter registry study (30), but similar to the 39.7% of severe disease in CLL patients on treatment or with treatment during the last year before the Omicron pandemic (26).

The rate of severe COVID-19 is influenced by vaccination and antiviral medication. Low seroconversion rates (20% - 30%) after COVID-19 RNA vaccination were observed in CLL patients and those under active BTKi treatment (31, 32). Unfortunately, we have no information on seroconversion rates in our patients after vaccination with the Chinese vaccine. Anyway, the vaccination rate in our patients was low. Antiviral treatment with NMVr within five days of developing symptoms reduces the incidence of severe COVID-19 in high-risk patients (33, 34). In our study, only a minority of CLL patients (18/82 [21.95%] on BTKi and 3 of 25 patients [12%] without treatment received NMVr within 5 days of developing early symptoms of COVID-19.

Progression of lymphadenopathy and splenomegaly were observed with the COVID-19-related interruption of BTKi treatment. Longer interruption of BTKi seemed associated with a higher risk of progression in our cohort. Progression of symptoms typical of CLL occurred within 10 days after BTKi cessation in some patients.

There are limitations to our study. First, this is a retrospective analysis relying on reports of patients and their caregivers. We cannot exclude that subjective perception of symptoms introduced bias in the analysis. However, we mostly analyzed objective parameters (hypoxemia, admission to ICU, survival) which hardly can be influenced by individual perception. Second, the number of cases, particularly in the no treatment group, was rather small reflecting day-to-day practice in a tertiary hospital in our country. Third, the previous lack of exposure to SARS-CoV-2, the low rates of vaccination with different vaccines, and the late use of antiviral treatment may have changed to rate and severity of COVID-19 compared with such events in the Western world. Our observation in Chinese patients therefore give a different but probably more “clean” view on the consequences of SARS-CoV-2 infection inherent to the properties of the virus variant.

## 5 Conclusion

Our results show that continuation of BTKi treatment in Chinese patients with CLL results in infection rates similar to those observed in untreated patients; however, the rates of severe COVID-19 were significantly higher in patients treated with BTKi. These observations probably apply also to patients living in other parts of the world although absolute numbers and frequencies of infection may be different because vaccines and vaccination policies may vary. Interruption of BTKi treatment during times of high prevalence/incidence of SARS-CoV-2 infections is recommended.

## Data availability statement

The raw data supporting the conclusions of this article will be made available by the authors, without undue reservation.

## Ethics statement

The studies involving humans were approved by Peking University Peoples’ Hospital ethics committee. The studies were conducted in accordance with the local legislation and institutional requirements. The participants provided their written informed consent to participate in this study.

## Author contributions

SY: Conceptualization, Data curation, Formal analysis, Funding acquisition, Investigation, Methodology, Project administration, Resources, Software, Supervision, Validation, Visualization, Writing – original draft, Writing – review & editing. RW: Writing – review & editing. HS: Writing – review & editing. YW: Writing – review & editing. YL: Writing – review & editing. XZ: Writing – review & editing. JL: Writing – review & editing. NS: Methodology, Writing – original draft, Writing – review & editing.
