# Supplementary material for: The impact of Bruton’s tyrosine kinase inhibitor treatment on COVID-19 outcomes in Chinese patients with chronic lymphocytic leukemia
Source: Front Oncol. 2024 May 21;14:1396913. doi: 10.3389/fonc.2024.1396913 (PMC11148257; doi:10.3389/fonc.2024.1396913)
Supplement: Supplementary file 1 [file DataSheet_1.docx]

Questionnaire for the BTKi on COVID-19 Outcomes in Patients with CLL

1. ID
2. Date of birth: Month Date Year
3. Sex: 0=Female, 1=Male
4. Date of the last visit: Month Date Year
5. Date of diagnosis: Month Date Year
6. The province of residence
7. Previous CLL treatment history:

0= none;

1=BTKi;

2= chemotherapy or immunochemotherapy

1. Current treatment:

0=none;

1=ibrutinib;

2=acalabrutinib;

3=zanubrutinib;

4=orelabrutinib;

5=chemoimmunotherapy;

6=venetoclax;

7=venetoclax combined with BTKi with or without obinutuzumab

1. Date of the first-line treatment: Month Date Year
2. Date of the current treatment: Month Date Year
3. Disease status before COVID-19:
   1. Time of doubling lymphocyte <6 months with a baseline absolute lymphocyte count ≥30x10^9^/L: Yes or no
   2. Lymph nodes >10cm: Yes or no
   3. Hemoglobin <10g/dl: Yes or no
   4. Platelet <100x10^9^/L: Yes or no
   5. Splenomegaly >6cm: Yes or no
4. Concurrent medical history:
   1. Diagnosis：
   2. Undertreatment：Yes or no
   3. Impact on health:

0=none,

1=interferes with normal activity,

2=disabling，

3=life threatening

1. Doses of SARS-Cov-2 vaccine:

0,

1,

2,

≥3

14. If vaccinated, then:

Date of the last dose

15 COVID-19 disease：Yes or no

If yes，then:

15.1 Number of previous infection episodes

15.2 Date of COVID-19 occurrence

15.3 Symptom of COVID-19 disease: Yes or no

15.4 Evidence of SARS-Cov-2 infection: 1=nuclear acid, 2=antigen examination

15.5 Hospitalization for COVID-19: Yes or no

15.6 Antivirus treatment: 0=none, 1= nirmatival/ritonaval; 2=monolaval

15.7 Pneumonia with evidence of X-ray or CT scan: Yes or no

15.8 Hypoxemia (SaO2 <93%): Yes or no

15.9 Necessary of oxygen supplement: Yes or no

15.10 ICU admission: Yes or no

15.11 Ventilator: 0=no, 1=Non-invasive, 2=Intubation

15.12 Death: Yes or no

If yes, then:

- - 1. Date of death
    2. Cause of death

15.13 Disruption of CLL treatment: Yes or no

If yes, then:

- - 1. Duration of disruption
    2. The reasons for the disruption:

0=contraindication of antivirus medicine

1=severe infection

2=decision of the patients

3=decision of the caregivers

4=decision of the physicians

- - 1. Changes of CLL associated symptom or laboratory findings after BTKi holding:
       1. Lymph nodes: 0=no change, 1= shrank, 2=enlargement
       2. Spleen: 0=no change, 1= shrank, 2=enlargement
       3. Absolute lymphocyte count: 0=no change, 1=decrease, 2=increase
  1. BTKi rechallenging after stopping: Yes or no

If yes, then

- - 1. Changes of CLL associated symptom or laboratory findings after BTKi rechallenging:
       1. Lymph nodes: 0=no change, 1= shrank, 2=enlargement
       2. Spleen: 0=no change, 1= shrank, 2=enlargement
       3. Absolute lymphocyte count: 0=no change, 1=decrease, 2=increase
