# Supplementary material for: The impact of Bruton’s tyrosine kinase inhibitor treatment on COVID-19 outcomes in Chinese patients with chronic lymphocytic leukemia
Source: Front Oncol. 2024 May 21;14:1396913. doi: 10.3389/fonc.2024.1396913 (PMC11148257; doi:10.3389/fonc.2024.1396913)
Supplement: Supplementary file 2 [file Table_1.docx]

**Supplementary Table 2:** Characteristics of patients who did not survive COVID-19

| ID number | “1” | “5” | “94” | “103” |
| --- | --- | --- | --- | --- |
| Sex | Male | Male | Female | Female |
| Age, years | 72 | 86 | 68 | 70 |
| CIRS | 3 | 5 | 0 | 2 |
| Time from diagnosis to first line treatment, months | 0.5 | 14.6 | 67.8 | 1.0 |
| Disease status | SD | SD | PD | SD |
| Vaccination | None | None | None | None |
| Lines of BTKi treatment | 1^st^ | 2^nd^ | 1^st^ | 2^nd^ |
| BTKi | Ibrutinib | Ibrutnib | Zanubrutinib | Orelabrutinib |
| Time on BTKi at COVID-19 diagnosis, months | 26 | 45 | 29 | 48 |
| Date of COVID-19 diagnosis | 2022/12/10 | 2022/12/7 | 2022/12/10 | 2022/12/10 |
| City of COVID-19 diagnosis | Beijing | Beijing | Wuhan, Hubei | Beijing |
| Duration between COVID-19 diagnosis to death, days | 50 | 53 | 31 | 29 |
| Pneumonia | yes | yes | yes | yes |
| Hypoxemia | yes | yes | yes | yes |
| Ventilation | No | Yes | No | No |
| Place of death | Emergency unit | ICU | Home | Home |
| Anti-virus treatment | None | NMVr for 5 days | None | None |

**Abbreviations:** BTKi, Bruton tyrosine kinase inhibitor; CIRS, Cumulative Illness Rating Scale; ICU, intensive care unit; NMVr, nirmatrelvir ritonavir; PD, progressive disease; SD, stable disease.
